# Temporal association between influenza A and B positivity and fever-clinic visit burden in Shanghai, China, 2022–2025: a retrospective time-series study

**DOI:** 10.3389/fpubh.2026.1912599

**Published:** 2026-09-15

**Authors:** Shan Yu, Tongtong Xu, Cunzheng Song, Xiaosong Chen, Yueqiu Gao

**Affiliations:** 1 Shanghai University of Traditional Chinese Medicine, Shanghai, China; 2 Renji Hospital, Shanghai Jiao Tong University School of Medicine, Shanghai, China

**Keywords:** fever clinic, healthcare utilization, hospital preparedness, influenza A, influenza B, respiratory virus surveillance

## Abstract

**Background:**

Temporal changes in respiratory virus circulation after the COVID-19 pandemic have created new challenges for fever-clinic preparedness. Outpatient influenza laboratory activity may provide useful operational information, but its type-specific temporal relationship with subsequent fever-clinic burden has not been well characterized.

**Methods:**

We conducted a retrospective time-series study using aggregated fever-clinic visit records and outpatient influenza testing data from Renji Hospital, Shanghai, China, from 2022 to 2025. Lead–lag correlations were evaluated over a primary −28 to +28-day window using 7-day centered moving averages, with trailing moving averages and an extended −56 to +56-day window as sensitivity analyses. High-burden days were defined as days with visits at or above the 90th percentile. Adjusted quasi-Poisson models accounted for day of week, calendar month, calendar year, and testing volume. Additional analyses excluded 2022, evaluated adjacent 0–21-day influenza A leads, and used Newey–West autocorrelation-robust inference.

**Results:**

We included 254,346 fever-clinic visits and 303,597 outpatient influenza testing episodes. Overall influenza positivity was 33.96%, with 84,819 influenza A-positive and 18,504 influenza B-positive episodes. Influenza A was the main contributor to increased overall activity, particularly in 2023 and 2025. Peak correlations occurred at −12 days for overall influenza positivity and −15 days for influenza A positivity, whereas influenza B peaked at the positive boundary of the primary window. In the extended analysis, the influenza B peak shifted to +50 days, indicating no stable leading relationship. High-burden days were preceded by higher overall influenza and influenza A positivity than reference days. In the conventional testing-volume-adjusted model, influenza A positivity at a 15-day lead was associated with daily fever-clinic visits, but this association was attenuated after exclusion of 2022 and was no longer statistically significant with Newey–West inference. Adjacent-lag analyses did not identify a robust positive day-specific association after false discovery rate correction.

**Conclusion:**

Outpatient influenza A positivity showed clearer temporal alignment with subsequent fever-clinic burden than influenza B. Although the fixed 15-day estimate was sensitive to period restriction and residual serial correlation, influenza A positivity may still serve as a complementary hospital-level surveillance indicator rather than a stand-alone forecasting tool.

## 1 Introduction

Influenza has historically been a major cause of acute respiratory illness worldwide, contributing to a substantial public health burden through its effects on morbidity, healthcare-seeking behavior, and medical resource consumption. A rise in influenza and other respiratory virus circulation can lead to increased demand for fever clinics and emergency departments, placing strain on frontline healthcare capacity (1). However, after the coronavirus disease 2019 (COVID-19) pandemic and the subsequent transition in public health measures, the epidemiology of respiratory pathogens has been markedly reconfigured, and previously stable seasonal expectations may no longer fully apply in the post-pandemic era (2). This makes it increasingly important to understand how influenza activity relates to real-world healthcare demand, both for clinical operations and for health-system preparedness.

Fever clinics serve as a frontline entry point for evaluating, triaging, and initially managing patients with respiratory symptoms, while also playing a critical role in monitoring respiratory infections, especially newly emerging or re-emerging respiratory pathogens (3). Fever-clinic consultation patterns reflect not only community respiratory disease activity but also changes in healthcare-system pressure, thus serving as a timely operational indicator of service burden (4). In China, national surveillance systems incorporate fever clinic consultation patterns, providing valuable macro-level epidemiological information and supporting national-level risk assessment. However, their design focus on broad, aggregated trends means they do not typically capture the day-to-day, locality-specific fluctuations that frontline clinical practice and regional health planning often require. This gap can be filled by long-term continuous monitoring at the fever-clinic level. Quantifying the temporal relationship between influenza activity and fever-clinic visits is essential for anticipating and mitigating surge-related stress on frontline settings.

This temporal relationship may have become more complex during the COVID-19 transition and subsequent period, as patterns of respiratory epidemiology showed notable shifts (5). Changes in pathogen transmission intensity, population immunity, healthcare-seeking behavior, and testing practice may together have reshaped both the seasonality and year-to-year variation of fever-clinic burden (6). At the same time, the relationship between influenza activity and clinical service demand may involve a lead–lag rather than purely contemporaneous structure, with potential operational relevance. Previous studies have focused largely on emergency surveillance during the pandemic or on the clinical characteristics of individual pathogens (7). Institution-level analyses describing the long-term dynamics of fever-clinic burden during the COVID-19 transition and subsequent period remain limited, particularly regarding the temporal relationship between influenza circulation and fever-clinic utilization. Whether influenza A and influenza B differ in their seasonal distribution, epidemic duration, and temporal association with healthcare demand has not been fully clarified.

Therefore, we performed a systematic retrospective analysis using continuous fever-clinic visit data and outpatient influenza laboratory testing records from a single hospital system covering January 2022 to December 2025. We aimed to characterize the dynamic pattern of fever-clinic burden during the COVID-19 transition and subsequent period, describe seasonal and interannual changes in influenza activity, and quantify the temporal alignment between fever-clinic visits and outpatient influenza positivity. These findings provide real-world evidence to inform hospital-level respiratory-virus surveillance, improve surge preparedness, and support operational planning for respiratory-virus-related service burden.

## 2 Materials and methods

### 2.1 Study design

This was a retrospective time-series study conducted at Renji Hospital, Shanghai Jiao Tong University School of Medicine, Shanghai, China, between January 1, 2022 and December 31, 2025. The dataset included fever-clinic visit data and outpatient influenza laboratory testing records across all hospital campuses. The fever clinics operate 24 h per day, 7 days per week, and primarily serve adult patients with febrile illness or respiratory symptoms. The unit of analysis was the calendar day. The study protocol was approved by the Institutional Ethics Committee of Renji Hospital (approval No. KY2024-113-B-RE-01).

### 2.2 Data sources and definitions

Daily data were extracted from the hospital electronic medical record system and influenza laboratory testing records. Influenza testing episodes were derived from outpatient laboratory records, including tests ordered in fever clinics and general outpatient clinics; emergency-department and inpatient tests were excluded. The extracted variables included: (1) daily fever-clinic visit counts; (2) daily numbers of influenza testing episodes; (3) daily numbers of influenza A-positive episodes; (4) daily numbers of influenza B-positive episodes; and (5) daily numbers of influenza A/B co-positive episodes.

Throughout the study period, the influenza testing platform and institutional laboratory workflow remained unchanged. Influenza testing was performed as part of routine clinical care rather than according to a study-mandated testing schedule. Therefore, testing decisions may have varied according to physician judgment, patient presentation, circulating respiratory pathogens, and healthcare-seeking behavior. To partially account for variation in testing intensity, daily influenza testing volume was included as an adjustment variable in the regression analyses.

Apparent duplicate laboratory records were identified and removed based on patient identifier, report date, and identical influenza A/influenza B test results. After deduplication, records were aggregated by calendar day to generate the analytic dataset, including daily fever-clinic visit counts, influenza testing episodes, influenza A-positive episodes, influenza B-positive episodes, and influenza A/B co-positive episodes. The aggregated dataset was independently checked by two investigators for accuracy and completeness. Data quality checks included assessment of date completeness, duplicate calendar dates, invalid denominators, and internal consistency among influenza A-positive episodes, influenza B-positive episodes, co-positive episodes, and overall influenza-positive episodes.

Overall influenza positivity was defined as positivity for either influenza A or influenza B. Influenza A/B co-positive episodes were counted once in the overall influenza-positive count but were included in both the influenza A-positive and influenza B-positive counts. Therefore, the number of overall influenza-positive episodes was calculated as influenza A-positive episodes plus influenza B-positive episodes minus influenza A/B co-positive episodes. Positivity rate was calculated as the number of positive episodes divided by the total number of influenza testing episodes. Monthly positivity rates were calculated using monthly aggregated positive episodes divided by monthly aggregated testing episodes, rather than by averaging daily positivity rates.

### 2.3 Statistical analysis

Data verification, analysis, and visualization were performed using Python 3.13.5. Continuous variables were summarized using medians with interquartile ranges (IQRs), and categorical or count variables were summarized using counts and percentages. All tests were two-sided, and *p* < 0.05 was considered statistically significant unless otherwise specified.

#### 2.3.1 Descriptive and monthly aggregation analyses

Annual totals of fever-clinic visits, influenza testing episodes, influenza A-positive episodes, influenza B-positive episodes, overall influenza-positive episodes, and influenza A/B co-positive episodes were calculated to describe the study population and laboratory testing burden.

For temporal analysis, monthly fever-clinic visit counts were summed from daily data. Monthly influenza positivity rates were derived from monthly aggregated positive episodes divided by monthly aggregated testing episodes. These monthly data were used to describe temporal trends in fever-clinic visits and influenza activity and to generate heatmaps showing seasonal and interannual patterns of influenza A and influenza B positivity.

#### 2.3.2 Lead–lag correlation analysis

Lead–lag correlation analysis was performed to evaluate the temporal relationship between influenza positivity and fever-clinic visits over a primary lag window of −28 to +28 days. Negative lag values indicated that influenza positivity preceded fever-clinic visits, whereas positive lag values indicated that influenza positivity followed fever-clinic visits.

To reduce day-of-week variation in this retrospective analysis, both fever-clinic visit counts and influenza positivity series were smoothed using 7-day centered moving averages. Because centered smoothing incorporates adjacent observations, including subsequent days, the lead–lag correlation results were interpreted as retrospective temporal associations and were used for temporal association screening rather than validation of real-time early-warning performance.

Pearson correlation coefficients were calculated between smoothed fever-clinic visits and smoothed overall influenza, influenza A, and influenza B positivity at each lag, using lag-specific complete-case pairs. The peak lag was defined as the lag day with the maximum positive correlation coefficient. Approximate 95% confidence intervals were calculated using Fisher’s z transformation. *p* values were adjusted for multiple lag comparisons using the Benjamini–Hochberg false discovery rate (FDR) method.

As sensitivity analyses, the lead–lag correlations were repeated using 7-day trailing moving averages and an extended −56 to +56-day lag window. The extended analysis was specified during revision to assess whether the influenza B maximum at +28 days in the primary window represented a boundary artifact.

#### 2.3.3 High-burden fever-clinic day analysis

High-burden fever-clinic days were defined using the 90th percentile of daily fever-clinic visits during the entire study period. Days with fever-clinic visits at or above this threshold were classified as high-burden days, and days below this threshold were classified as non-high-burden reference days.

To describe influenza activity before high-burden fever-clinic days, event-centered mean positivity profiles for overall influenza, influenza A, and influenza B were calculated from 28 days before each index day to the index day itself, corresponding to lag days −28 to 0. The same procedure was applied to non-high-burden reference days for comparison. This analysis was intended to provide a descriptive operational comparison rather than a formal prediction model.

#### 2.3.4 Adjusted regression analysis

To evaluate whether influenza positivity was associated with daily fever-clinic visits after adjustment for calendar patterns and testing intensity, quasi-Poisson regression models with a log link were fitted with daily fever-clinic visits as the outcome.

In this exploratory two-step analysis, exposure lags for the adjusted regression models were selected from the descriptive lead–lag correlation profiles. The evaluated lags were overall influenza positivity at a 12-day lead, influenza A positivity at a 15-day lead, and influenza B positivity at lag 0 as an exploratory contemporaneous comparison. Influenza B at lag 0 was evaluated because influenza B did not show a stable negative-lag peak in the descriptive lead–lag analysis.

Adjusted incidence rate ratios (IRRs) were reported per 10-percentage-point increase in influenza positivity. The seasonality-adjusted model included day of week, calendar month, and calendar year. The testing-volume-adjusted model additionally included lag-aligned log(1 + influenza testing episodes) to partially account for variation in testing intensity. Because the exposure lags were selected from the same dataset used for regression, these models were interpreted as exploratory adjusted-association analyses rather than confirmatory causal estimates or externally validated forecasting models.

#### 2.3.5 Sensitivity and robustness analyses

Sensitivity analyses were performed to assess temporal heterogeneity, residual serial correlation, and lag-selection uncertainty. First, the primary regression models were repeated after excluding 2022 outcome observations and restricting the outcome period to January 1, 2023 through December 31, 2025. Exposure lags were retained from the primary analysis. Lagged predictors were generated from the continuous time series before restriction, so that late-2022 observations were used only where required to construct early-2023 lagged exposures.

Second, residual serial correlation in the fully adjusted influenza A 15-day model was assessed using the lag-1 Pearson residual correlation and Ljung–Box tests at lags 7, 14, and 28 days. The same mean specification was then analyzed using heteroskedasticity- and autocorrelation-consistent Newey–West covariance estimates. A 7-day bandwidth was used as the primary HAC specification, with 14- and 28-day bandwidths examined additionally.

Third, an adjacent-lag sensitivity analysis evaluated influenza A leads from 0 to 21 days using the same fully adjusted model specification. HAC/Newey–West covariance estimates with a 7-day bandwidth were used for inference. *p* values were adjusted separately within the 2022–2025 and 2023–2025 analyses across the 22 evaluated lead times using the Benjamini–Hochberg false discovery rate procedure.

## 3 Results

### 3.1 Study population and influenza testing burden

A total of 254,346 fever-clinic visits across all hospital campuses were recorded, and 303,597 outpatient influenza testing episodes were included from available laboratory records. Overall, 84,819 episodes (27.94%) tested positive for influenza A, and 18,504 episodes (6.10%) for influenza B. The overall influenza positivity rate, defined as influenza A or B positivity, was 33.96% (103,093 episodes) (Table 1).

**Table 1:** Study overview of fever-clinic visits, outpatient influenza testing, and positivity, 2022–2025.

Year	Fever-clinic visits, n	Daily fever-clinic visits, median (IQR)	Influenza testing episodes, n	Daily influenza testing episodes, median (IQR)	Influenza A positive, n (%)	Influenza B positive, n (%)	Overall influenza positive, n (%)	Influenza A + B co-positive, n
2022	47,706	110 (61–148)	7,851	19 (2–31)	1,911 (24.34)	993 (12.65)	2,904 (36.99)	0
2023	103,813	236 (180–370)	103,255	225 (77–330)	46,918 (45.44)	3,241 (3.14)	50,040 (48.46)	119
2024	65,435	124 (87–223)	104,074	206 (124–368)	14,157 (13.60)	13,894 (13.35)	27,947 (26.85)	104
2025	37,392	87 (65–117)	88,417	170 (138–250)	21,833 (24.70)	376 (0.43)	22,202 (25.11)	7
Overall	254,346	121 (81–217)	303,597	145 (40–255)	84,819 (27.94)	18,504 (6.10)	103,093 (33.96)	230

Values are shown as n, median (IQR), or n (%). Positivity percentages were calculated using influenza testing episodes in the corresponding year as the denominator. Overall influenza positivity was defined as positivity for either influenza A or influenza B. Influenza A/B co-positive episodes were counted once in the overall influenza-positive count. IQR, interquartile range.

The highest annual fever-clinic visit count was observed in 2023, with 103,813 visits, followed by 2024, 2022, and 2025. Influenza testing volume was highest in 2024 and 2023. Influenza A positivity was highest in 2023 and remained prominent in 2025, whereas influenza B positivity was more prominent in 2022 and 2024. In 2024, the annual numbers of influenza A-positive and influenza B-positive episodes were similar, with 14,157 and 13,894 positive episodes, respectively.

### 3.2 Monthly trends in fever-clinic visits and influenza positivity

Monthly fever-clinic visits showed substantial temporal variation during the study period (Figure 1). Higher monthly fever-clinic visit counts were observed in late 2022 to early 2023, spring 2023, late 2023 to early 2024, early 2025, and late 2025. Periods with higher fever-clinic visit counts frequently coincided with or followed periods of increased overall influenza positivity, although the temporal patterns of visit counts and influenza positivity were not identical.

**Figure 1:**
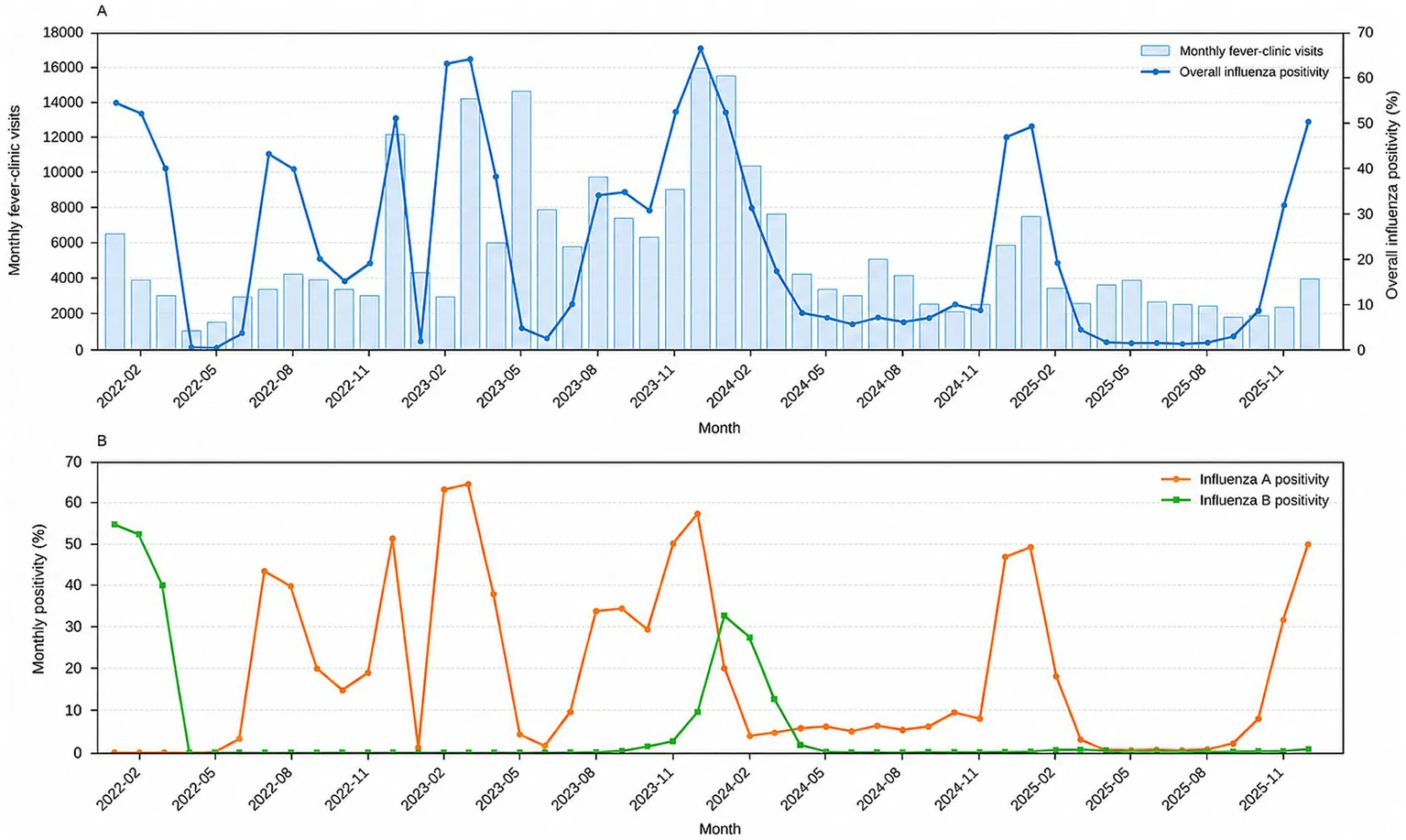
Monthly temporal patterns of fever-clinic visits and influenza activity, 2022–2025. Monthly positivity rates were calculated as aggregated monthly positive counts divided by aggregated monthly influenza testing episodes. Overall influenza positivity was defined as positivity for either influenza A or influenza B.

Overall influenza positivity showed recurrent increases during the study period. Influenza A positivity contributed to most periods of increased overall influenza positivity, particularly during 2023 and 2025. Influenza B positivity was more temporally restricted, with higher activity mainly observed in early 2022 and early 2024.

### 3.3 Seasonal and interannual patterns of influenza A and influenza B

The heatmaps further demonstrated heterogeneous seasonal and interannual patterns of influenza A and influenza B activity (Figure 2). Influenza A positivity was observed across multiple years, with higher monthly positivity in several winter or early-spring periods and in selected mid-to-late year months. Higher influenza A positivity was especially apparent in 2023 and 2025. Influenza B positivity showed a more time-limited pattern. Higher monthly influenza B positivity was mainly observed in early 2022 and early 2024, whereas influenza B positivity remained low in most other months.

**Figure 2:**
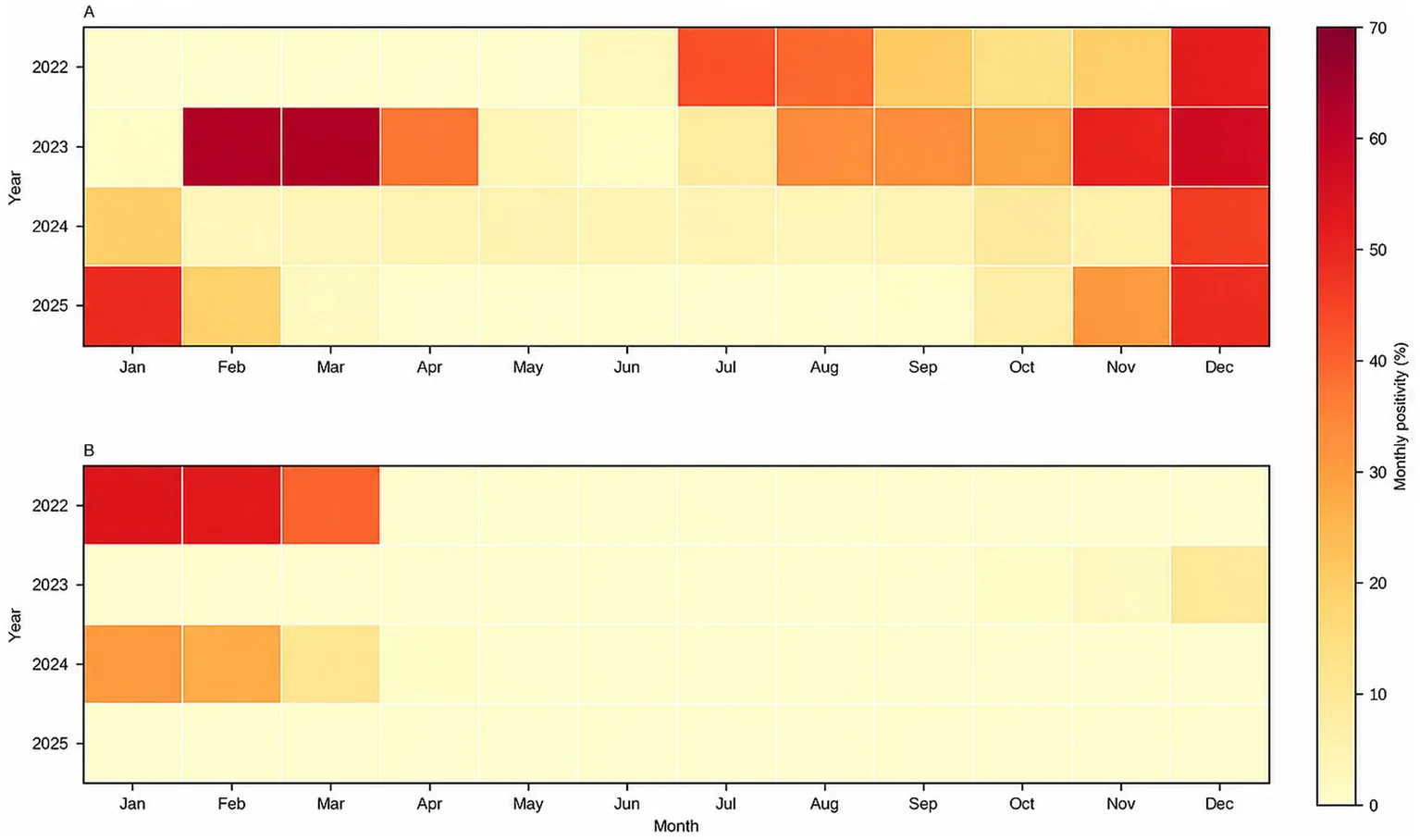
Seasonal and interannual patterns of Influenza A and Influenza B positivity, 2022–2025. Monthly positivity rates were calculated using aggregated influenza testing episodes as the denominator. Lighter colors indicate lower positivity, and darker red indicates higher positivity.

### 3.4 Lead–lag correlation analysis

Lead–lag correlation analysis was performed to assess the temporal alignment between influenza positivity and fever-clinic visits in the primary −28 to +28-day window (Figure 3 and Table 2). For overall influenza positivity, the maximum positive correlation with fever-clinic visits was observed at lag −12 days (*r* = 0.585; 95% CI, 0.550–0.618; FDR-adjusted *p* < 0.001). For influenza A positivity, the maximum positive correlation occurred at lag −15 days (*r* = 0.558; 95% CI, 0.522–0.593; FDR-adjusted *p* < 0.001). Broad positive correlation windows were observed for both overall influenza and influenza A positivity.

**Figure 3:**
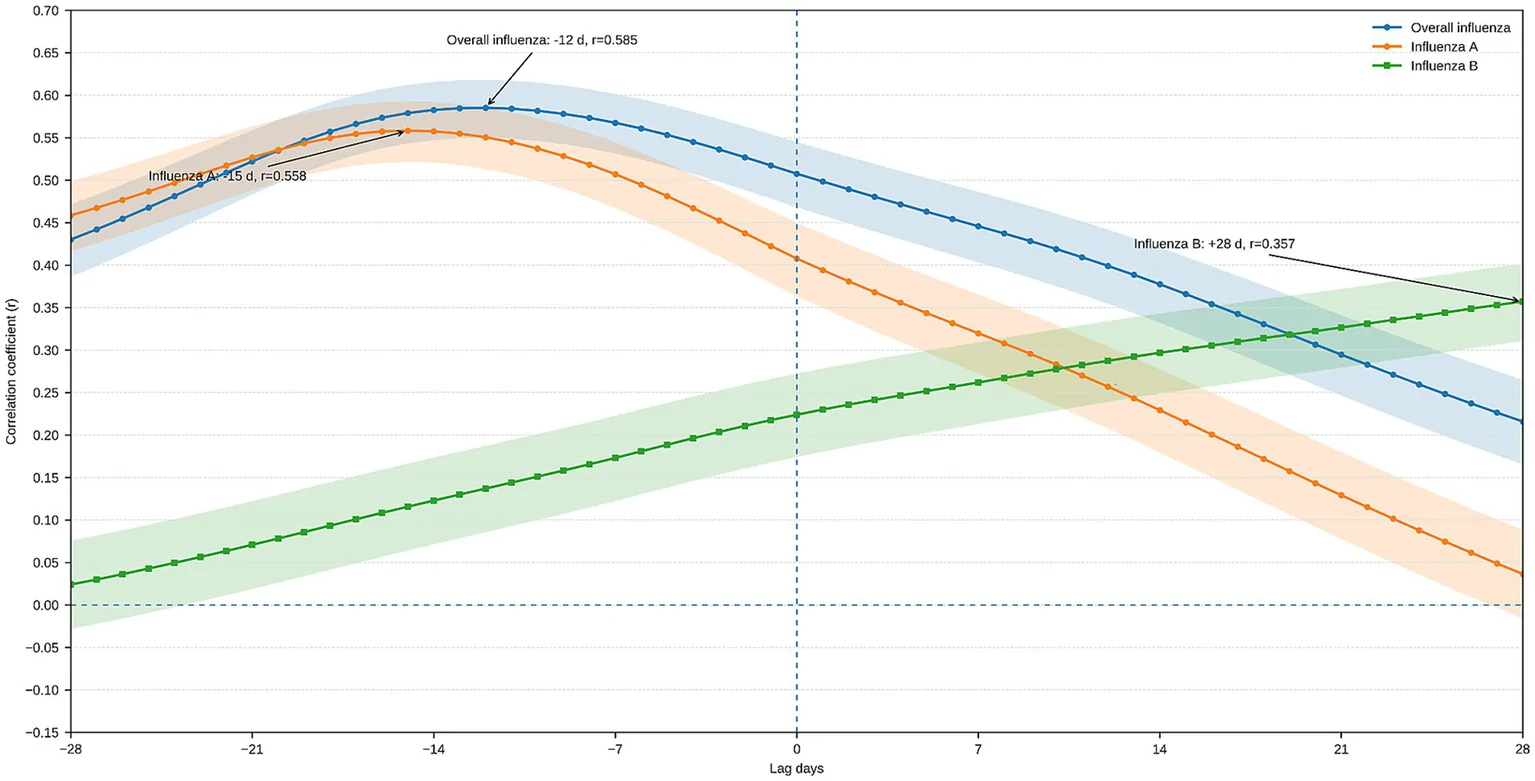
Lead–lag correlation profiles between influenza positivity and fever-clinic visits over −28 to +28 days. Negative lag values indicate that influenza positivity preceded fever-clinic visits, whereas positive lag values indicate that influenza positivity followed fever-clinic visits. Correlations were calculated using 7-day centered moving-average daily series. Shaded bands indicate approximate 95% confidence intervals. Overall influenza positivity was defined as positivity for either influenza A or influenza B.

**Table 2:** Peak lead–lag correlations between influenza positivity and fever-clinic visits in the primary −28 to +28-day lag window.

Pathogen	Peak lag, days	Peak correlation coefficient, r	Approximate 95% CI at peak	FDR-adjusted *p* value	FDR-significant window, days
Overall influenza positivity	−12	0.585	0.550–0.618	<0.001	−28 to +28
Influenza A positivity	−15	0.558	0.522–0.593	<0.001	−28 to +26
Influenza B positivity	+28	0.357	0.311–0.402	<0.001	−23 to +28

Lead–lag correlations were evaluated over the primary −28 to +28-day lag window using 7-day centered moving-average daily series. Negative lags indicate that influenza positivity preceded fever-clinic visits, whereas positive lags indicate that influenza positivity followed fever-clinic visits. Confidence intervals were calculated using Fisher’s z transformation. p values were adjusted using the Benjamini–Hochberg false discovery rate method. The FDR-significant window denotes the contiguous FDR-significant interval containing the maximum positive correlation. Because adjacent lagged observations are serially correlated, lag-specific inferential statistics should be interpreted descriptively. CI, confidence interval; FDR, false discovery rate.

For influenza B positivity, the maximum positive correlation in the primary window occurred at lag +28 days (*r* = 0.357; 95% CI, 0.311–0.402; FDR-adjusted *p* < 0.001), at the boundary of the evaluated lag window (Table 2). In the extended −56 to +56-day analysis, the influenza B maximum shifted to +50 days (*r* = 0.412; 95% CI, 0.368–0.455; FDR-adjusted *p* < 0.001), whereas the overall influenza and influenza A maxima remained at −12 and −15 days, respectively (Supplementary Figure S1 and Supplementary Table S6). This pattern did not support a stable leading relationship between influenza B positivity and fever-clinic visits in this hospital-level dataset.

Sensitivity analysis using 7-day trailing moving averages preserved the principal lag structure observed with centered smoothing, with peak lags of −12 days for overall influenza, −15 days for influenza A, and +28 days for influenza B in the primary window (Supplementary Table S2).

### 3.5 Influenza positivity profiles before high-burden fever-clinic days

Using the *P*90 threshold of 377 visits per day, 147 high-burden fever-clinic days were identified. Event-centered descriptive profiles of influenza positivity from 28 days before the index day to the index day were compared between high-burden days and non-high-burden reference days (Figure 4 and Supplementary Table S1). Overall influenza positivity was consistently higher for high-burden days than for reference days across the plotted window. At lag −28 days, mean overall influenza positivity was 38.20% before high-burden days compared with 18.85% before reference days. At lag −7 days, the corresponding values were 45.14 and 18.58%, respectively. On the index day, mean overall influenza positivity was 41.57% for high-burden days and 19.12% for reference days. The largest absolute difference was observed at lag −14 days.

**Figure 4:**
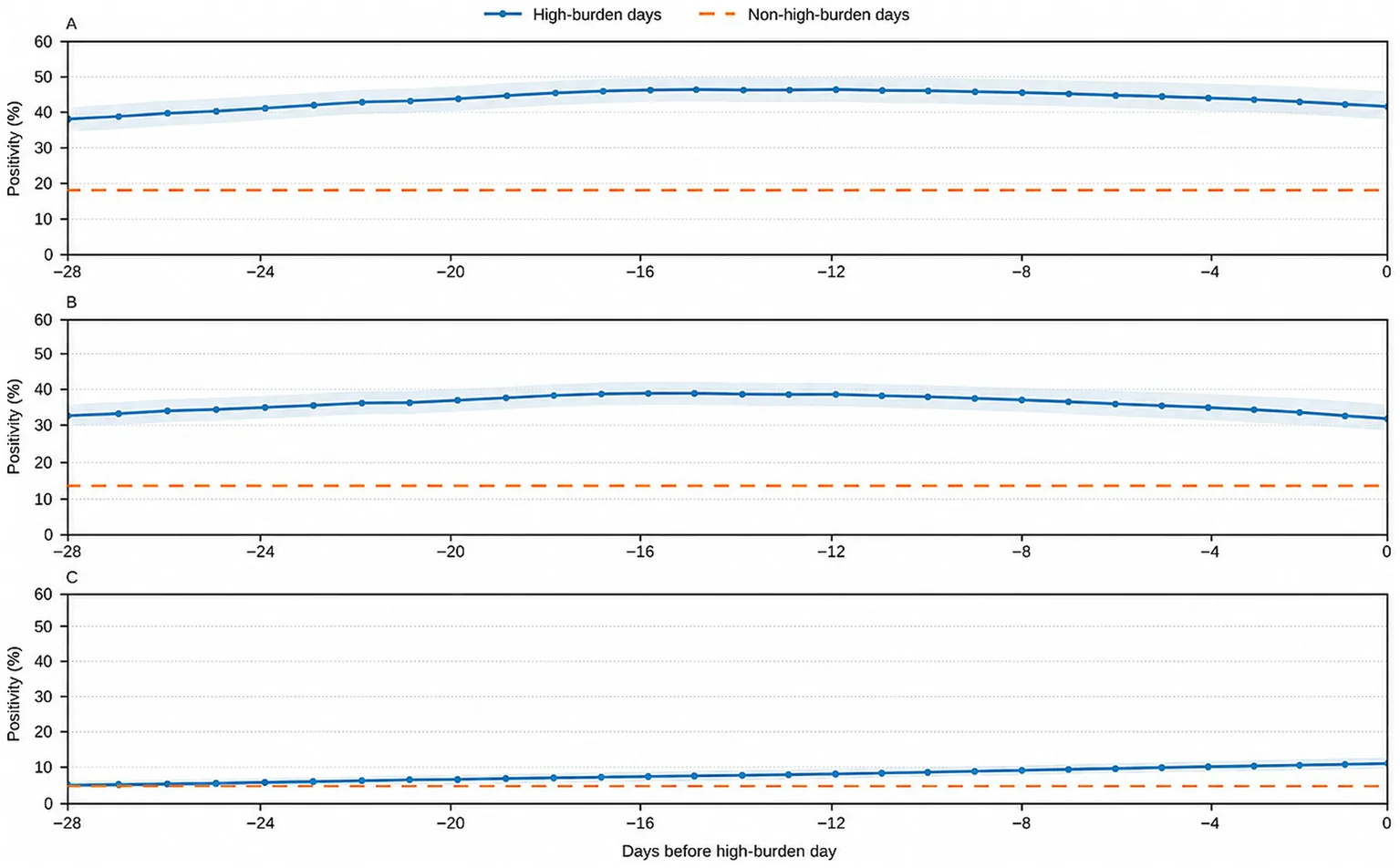
Influenza positivity before high-burden fever-clinic days, 2022–2025. High-burden days were defined as fever-clinic visit days at or above the 90th percentile, corresponding to ≥377 daily visits. Curves show mean daily positivity from 28 days before the index day to the index day. Shaded bands indicate approximate 95% confidence intervals. Overall influenza positivity was defined as positivity for either Influenza A or Influenza B.

Influenza A positivity showed a similar descriptive pattern. At lag −28 days, mean influenza A positivity was 33.15% before high-burden days compared with 14.02% before reference days. At lag −7 days, the corresponding values were 36.50 and 14.23%, respectively. The largest absolute difference for influenza A was observed at lag −14 days. For influenza B positivity, absolute differences between high-burden and reference days were smaller, ranging from 0.32 to 5.94 percentage points across lags. These event-centered descriptive analyses indicate that overall influenza and influenza A positivity were already elevated during the 28 days preceding high-burden fever-clinic days, whereas influenza B positivity showed only limited differences between high-burden and reference days.

### 3.6 Adjusted association between lagged influenza positivity and fever-clinic visits

In conventional daily quasi-Poisson regression models, influenza positivity measures were associated with fever-clinic visit burden in seasonality-adjusted analyses. After further adjustment for lag-aligned influenza testing volume, influenza A positivity at a 15-day lead remained associated with daily fever-clinic visits (IRR, 1.040; 95% CI, 1.024–1.055; *p* < 0.001), whereas the corresponding associations for overall influenza positivity at a 12-day lead and influenza B positivity at lag 0 were attenuated after testing-volume adjustment and were no longer statistically significant (Table 3). These conventional estimates did not account for residual serial correlation, which was evaluated in subsequent sensitivity analyses.

**Table 3:** Conventional adjusted quasi-Poisson regression models for the association between lagged influenza positivity and daily fever-clinic visits.

Model	Exposure	Lag used	Adjusted IRR (95% CI)	*p* value	N days
Seasonality-adjusted	Overall influenza positivity	12-day lead	1.152 (1.136–1.168)	<0.001	1,403
Seasonality-adjusted	Influenza A positivity	15-day lead	1.130 (1.115–1.146)	<0.001	1,400
Seasonality-adjusted	Influenza B positivity	0-day exploratory	1.127 (1.098–1.156)	<0.001	1,415
Seasonality + testing-volume adjusted	Overall influenza positivity	12-day lead	1.011 (0.993–1.030)	0.229	1,403
Seasonality + testing-volume adjusted	Influenza A positivity	15-day lead	1.040 (1.024–1.055)	<0.001	1,400
Seasonality + testing-volume adjusted	Influenza B positivity	0-day exploratory	0.984 (0.960–1.009)	0.203	1,415

Adjusted incidence rate ratios (IRRs) were estimated using quasi-Poisson regression with daily fever-clinic visits as the outcome and are expressed per 10-percentage-point increase in influenza positivity. Seasonality-adjusted models included day of week, calendar month, and calendar year. Testing-volume-adjusted models additionally included lag-aligned log(1 + influenza testing episodes). These conventional estimates do not account for residual serial correlation; autocorrelation-robust sensitivity analyses are presented in Supplementary Table S4. CI, confidence interval; IRR, incidence rate ratio.

### 3.7 Sensitivity analyses

The influenza A 15-day association was sensitive to restriction of the study period. After exclusion of 2022, the fully adjusted estimate for influenza A at the 15-day lead was attenuated and was no longer statistically significant (IRR, 0.984; 95% CI, 0.966–1.003; *p* = 0.090), indicating period sensitivity (Supplementary Table S3).

Residual diagnostics demonstrated substantial serial dependence in the primary fully adjusted influenza A model (lag-1 Pearson residual correlation, 0.868; Ljung–Box tests at lags 7, 14, and 28 days, all *p* < 0.001). With 7-day Newey–West HAC standard errors, the influenza A 15-day estimate was no longer statistically significant (IRR, 1.040; 95% CI, 0.990–1.092; *p* = 0.119); conclusions were unchanged with 14- and 28-day HAC bandwidths (Supplementary Table S4).

Adjacent-lag analyses across 0–21-day influenza A leads using 7-day HAC-robust inference did not identify a uniquely robust positive day-specific association after FDR correction (Supplementary Table S5). Together, these findings indicate that influenza A positivity showed descriptive temporal alignment with fever-clinic burden, whereas the fixed 15-day adjusted association should be interpreted as exploratory rather than confirmatory.

## 4 Discussion

This retrospective time-series study based on fever-clinic visit records and influenza laboratory testing data from 2022 to 2025 demonstrated that both fever-clinic burden and influenza activity exhibited clear temporal variation. Overall influenza positivity increased repeatedly during the study period, with influenza A being the major component of most periods of increased overall influenza activity, particularly in 2023 and 2025. In contrast, influenza B activity was more temporally restricted, mainly concentrated in early 2022 and early 2024. The lead–lag analysis further revealed a clearer temporal association between influenza A positivity and fever-clinic visit burden, whereas the correlation for influenza B was weaker and its temporal pattern was less stable. These findings suggest that influenza A positivity may serve as a hospital-level contextual signal for operational preparedness, although it should not be interpreted as a fixed forecasting interval.

### 4.1 Epidemiological context of influenza activity during 2022–2025

Compared with the winter–spring single-peak or double-peak influenza patterns commonly observed in China before the COVID-19 pandemic (8), fever-clinic visits and influenza activity in this study showed different temporal features. These findings should therefore be interpreted within the broader reconfiguration of respiratory-virus circulation during the COVID-19 transition and post-transition period. The recurrent increases in influenza positivity observed here may reflect both the recovery of influenza transmission and changes in population immunity and healthcare-seeking behavior (9). Global and domestic studies have reported altered influenza circulation after the pandemic, including alternating activity of influenza A and influenza B (10). A multi-state analysis by the US CDC found that influenza B/Victoria rebounded before influenza A in the 2021–2022 season, whereas the 2022–2023 season shifted to A(H3N2) dominance (11). European studies have similarly reported out-of-season influenza activity, including unusual summer outbreaks (12). Consistent with these observations, a Shanghai study demonstrated phased recovery of influenza B and influenza A activity during and after the COVID-19 pandemic (13), which aligns with our finding of active influenza B in early 2022 and more prominent influenza A activity in 2023 and 2025. Thus, this study describes not only the operational characteristics of fever-clinic burden in a large urban hospital, but also provides supplementary real-world evidence of altered respiratory-virus circulation after COVID-19-related disruptions.

Of note, 2022 represented an epidemiologically and operationally distinct period in Shanghai. Extensive SARS-CoV-2 Omicron circulation and stringent public-health restrictions may have affected population mobility, healthcare-seeking behavior, fever-clinic utilization, and clinically driven respiratory-virus testing. Consistent with this context, influenza testing volume and laboratory-confirmed influenza detections were substantially lower in 2022 than in subsequent years. The attenuation of the fixed 15-day lead association for influenza A after restricting the analysis to 2023–2025 suggests that part of the full-period regression signal was influenced by cross-period heterogeneity. We therefore interpret the 2022–2025 analysis as describing the complete COVID-19 transition and post-transition period, whereas the 2023–2025 restricted analysis provides an assessment of temporal robustness rather than evidence that the 2022 data were invalid.

### 4.2 Lead–lag association between influenza A and fever-clinic burden

The following discussion focuses on the relationship between influenza A positivity and fever-clinic burden, because influenza A was the main contributor to recurrent increases in overall influenza activity during the study period. The temporal association between influenza activity and fever-clinic visits is clinically plausible. Influenza infection commonly causes fever, cough, sore throat, myalgia, and other symptoms, which may prompt patients to seek care at fever clinics (14). Previous studies have also reported temporal associations between influenza-like illness or laboratory-confirmed influenza activity and emergency department or fever-clinic visits (15). Similar attempts have been made using indirect data sources, such as Google Flu Trends, to estimate influenza-related healthcare utilization (16). Compared with such indirect indicators, our analysis was based on outpatient laboratory-confirmed influenza positivity and objective fever-clinic visit records, providing a more direct description of the relationship between influenza activity and hospital service demand.

The type-specific findings are also epidemiologically plausible. Influenza A and influenza B differ in transmission dynamics, population immunity, age distribution, and clinical presentation (17–19). Influenza A often shows greater epidemic potential than influenza B and may be more likely to cause broader epidemic waves (20). During the post-COVID-19 period, prolonged non-pharmaceutical interventions may also have reshaped population susceptibility to respiratory viruses and contributed to the resurgence of influenza activity after relaxation of control measures (21). In addition, previous studies have suggested age- and pathogen-specific differences in respiratory infection burden during the post-COVID-19 period (22). These factors may partly explain why influenza A showed clearer temporal alignment with fever-clinic visits, whereas influenza B showed a weaker and boundary-dependent pattern in the lead–lag analyses.

However, the adjusted regression results should be interpreted more cautiously than the descriptive lead–lag curves alone. In the conventional testing-volume-adjusted quasi-Poisson model, influenza A positivity at a 15-day lead was associated with daily fever-clinic visits, with an IRR of 1.040 per 10-percentage-point increase in positivity. This exact lag-specific estimate was not fully robust in sensitivity analyses. After restricting the analysis to 2023–2025, the estimate was attenuated and was no longer statistically significant. Similarly, after applying Newey–West heteroskedasticity- and autocorrelation-consistent standard errors, the 15-day estimate was no longer statistically significant. Adjacent-lag analyses across 0–21-day leads also did not identify a uniquely robust positive day-specific association after FDR correction. Therefore, the conventional quasi-Poisson result should not be taken as evidence of a fixed 15-day predictive effect. A more appropriate interpretation is that influenza A positivity showed an approximate 1–2-week temporal alignment with subsequent fever-clinic burden.

This hospital-level lag should also not be equated with the incubation period or duration of illness in an individual patient. It more likely reflects the combined effects of epidemic-wave progression, testing behavior, outpatient healthcare-seeking patterns, and accumulation of clinical service demand. In addition, the fever-clinic population in this study was predominantly adult, whereas children and adolescents may contribute earlier in community influenza transmission chains. Therefore, the observed influenza A lead–lag pattern is best understood as an institution-level operational association. It may provide contextual information for hospital preparedness, but it should not be interpreted as a generalizable community-level early-warning interval or as an externally validated forecasting rule.

### 4.3 Influenza positivity as an indicator of overall respiratory pressure

Another issue in interpreting these findings is that fever-clinic visits represent a broader healthcare-utilization outcome rather than the disease burden of a specific pathogen. In this study, some months recorded high fever-clinic visit volumes despite relatively low influenza positivity, suggesting that fever-clinic burden during the COVID-19 transition and subsequent period may have been jointly influenced by multiple respiratory pathogens, including respiratory syncytial virus, *Mycoplasma pneumoniae*, and SARS-CoV-2 (23–28). During the summer and autumn of 2022, out-of-season resurgences of RSV were reported in many countries (23). In the second half of 2023, a marked epidemic of *Mycoplasma pneumoniae* was reported in multiple regions of China, particularly among school-aged children, which may have contributed to broader respiratory-service pressure not captured by influenza positivity alone (24). During 2024–2025, continued circulation and lineage replacement of SARS-CoV-2 Omicron descendants reported in China may also have represented an unmeasured contributor to respiratory-infection-related fever-clinic demand (25). In addition, healthcare-seeking behavior, hospital triage policies, testing accessibility, and regional distribution of medical resources may also affect visit volume (29). Accordingly, the temporal association observed in this study should not be interpreted as evidence that influenza alone drove the increase in fever-clinic visits. A more appropriate interpretation is that influenza positivity, particularly influenza A positivity, represents a measurable component of broader respiratory-infection pressure that may help hospitals identify periods of increasing fever-clinic demand.

The influenza B findings also support this interpretation. In the primary −28 to +28-day window, the maximum correlation for influenza B occurred at the positive boundary of the evaluated lag window, and the extended −56 to +56-day analysis shifted the maximum further into the positive-lag direction. Because positive lag values indicate that influenza B positivity followed rather than preceded fever-clinic visits, these findings do not support influenza B positivity as a useful leading indicator for surge preparedness. The significant lag-0 association observed in the 2023–2025 restricted analysis is better interpreted as a contemporaneous relationship with service burden rather than as a prospective signal. Thus, influenza B may still contribute to respiratory-care demand during periods of circulation, but it did not show the prospective temporal structure observed for influenza A; the exact location of the long positive-lag peak should not be overinterpreted.

From an operational perspective, the potential value of the influenza A signal may lie in its timing rather than in a fixed forecasting horizon. An approximately 1–2-week interval may be practically useful for hospitals because it is long enough to review staffing and duty rosters, antiviral and diagnostic inventories, specimen-collection supplies, laboratory capacity, triage arrangements, and fever-clinic space before demand increases. At the same time, this interval remains close enough to the anticipated burden to be relevant to short-term operational decisions. Influenza surveillance may therefore complement broader respiratory-virus surveillance by informing preparedness for increasing service pressure (30).

### 4.4 Limitations and future directions

This study has several limitations. First, it was a retrospective study from a single hospital system, and the fever-clinic population was predominantly adult. Therefore, the observed lead–lag pattern should be interpreted as a hospital-level operational association rather than as the onset of community influenza circulation or a generalizable community-level early-warning interval. Second, 2022 represented an epidemiologically and operationally distinct period in Shanghai, and the attenuation of the fixed 15-day influenza A association after restricting the analysis to 2023–2025 indicates period sensitivity. The 2022–2025 analysis therefore describes the complete COVID-19 transition and post-transition period, whereas the restricted analysis provides an assessment of temporal robustness. Third, influenza positivity was derived from outpatient laboratory records, including fever-clinic and general outpatient testing, rather than from fever-clinic attendees alone. Although emergency-department and inpatient tests were excluded, and testing volume was adjusted for in the regression models, clinically driven testing and changes in healthcare-seeking behavior may still have influenced positivity estimates.

In addition, fever-clinic visits represent a broad healthcare-utilization outcome and cannot be attributed to influenza alone. Other respiratory pathogens, including respiratory syncytial virus, *Mycoplasma pneumoniae*, and SARS-CoV-2, were not incorporated as time-varying covariates and may have contributed to fever-clinic burden during the study period. Methodologically, the lead–lag analyses were retrospective, and centered moving averages do not represent real-time forecasting. The conventional quasi-Poisson regression was also sensitive to residual autocorrelation and period restriction; therefore, the fixed 15-day estimate should be interpreted as exploratory rather than confirmatory. Taken together, these limitations suggest that influenza A positivity is best viewed as a complementary surveillance signal for increasing respiratory-related service pressure, not as a stand-alone forecasting tool. Future studies should incorporate multi-pathogen laboratory time series, pediatric and community data, and prospective evaluation of whether surveillance-guided preparedness actions improve patient flow, resource allocation, or clinical outcomes.

## 5 Conclusion

Influenza A positivity showed an approximately 1–2-week temporal alignment with subsequent fever-clinic burden and may provide contextual information for hospital-level preparedness. However, the exact 15-day adjusted association was sensitive to study-period selection and residual serial correlation and should not be interpreted as a validated fixed forecasting point. Influenza B showed contemporaneous or trailing relationships but no useful leading signal. These findings support influenza A positivity as a complementary operational surveillance indicator alongside broader respiratory-pathogen and healthcare-utilization data.

## Data Availability

The original contributions presented in the study are included in the article/Supplementary material, further inquiries can be directed to the corresponding author/s.
