# Supplementary material for: Temporal association between influenza A and B positivity and fever-clinic visit burden in Shanghai, China, 2022–2025: a retrospective time-series study
Source: Front Public Health. 2026 Sep 15;14:1912599. doi: 10.3389/fpubh.2026.1912599 (PMC13619934; doi:10.3389/fpubh.2026.1912599)
Supplement: Supplementary file 1 [file Table_1.DOCX]

Supplementary Table S1. Influenza positivity before high-burden fever-clinic days, 2022–2025

| **Pathogen** | **Lag day relative to high-burden day** | **High-burden days, mean % (95% CI)** | **Non-high-burden days, mean % (95% CI)** | **Difference, percentage points** |
| --- | --- | --- | --- | --- |
| Overall influenza positivity | −28 | 38.20 (34.54–41.85) | 18.85 (17.69–20.01) | 19.35 |
| Overall influenza positivity | −21 | 43.19 (39.50–46.89) | 18.46 (17.33–19.58) | 24.73 |
| Overall influenza positivity | −14 | 46.29 (42.58–50.00) | 18.28 (17.18–19.38) | 28.01 |
| Overall influenza positivity | −7 | 45.14 (41.16–49.11) | 18.58 (17.48–19.68) | 26.56 |
| Overall influenza positivity | 0 | 41.57 (37.35–45.79) | 19.12 (18.00–20.23) | 22.45 |
| Influenza A positivity | −28 | 33.15 (29.80–36.50) | 14.02 (12.97–15.07) | 19.13 |
| Influenza A positivity | −21 | 36.82 (33.23–40.42) | 13.81 (12.79–14.82) | 23.02 |
| Influenza A positivity | −14 | 38.85 (34.99–42.71) | 13.78 (12.78–14.77) | 25.07 |
| Influenza A positivity | −7 | 36.50 (32.36–40.63) | 14.23 (13.23–15.23) | 22.26 |
| Influenza A positivity | 0 | 31.59 (27.32–35.86) | 14.95 (13.92–15.97) | 16.64 |
| Influenza B positivity | −28 | 5.16 (3.61–6.72) | 4.84 (4.09–5.59) | 0.32 |
| Influenza B positivity | −21 | 6.52 (4.71–8.32) | 4.66 (3.92–5.40) | 1.86 |
| Influenza B positivity | −14 | 7.58 (5.63–9.53) | 4.51 (3.79–5.24) | 3.07 |
| Influenza B positivity | −7 | 8.80 (6.70–10.89) | 4.35 (3.63–5.07) | 4.45 |
| Influenza B positivity | 0 | 10.12 (7.86–12.38) | 4.18 (3.47–4.88) | 5.94 |

Note: Values are mean daily positivity percentages with approximate 95% confidence intervals calculated as mean ± 1.96 × SE. Lags indicate days before the high-burden index day, with day 0 representing the index day. High-burden days were defined as fever-clinic visit days at or above the 90th percentile, corresponding to ≥377 visits/day; non-high-burden days were defined as <377 visits/day. Difference was calculated as the high-burden mean minus the non-high-burden mean in percentage points. Overall influenza positivity was defined as positivity for either influenza A or influenza B. These comparisons were descriptive. CI, confidence interval; SE, standard error.

**Supplementary Table S2. Sensitivity analysis of lead–lag correlations using 7-day trailing moving averages**

| **Positivity series** | **Peak lag, days** | **Peak correlation coefficient, r** | **95% CI at peak** | **FDR-adjusted *P* value at peak** | **FDR-significant lag window, days** | **N pairs at peak** |
| --- | --- | --- | --- | --- | --- | --- |
| Overall influenza positivity | −12 | 0.588 | 0.553–0.621 | <0.001 | −28 to +28 | 1,438 |
| Influenza A positivity | −15 | 0.562 | 0.525–0.596 | <0.001 | −28 to +26 | 1,435 |
| Influenza B positivity | +28 | 0.349 | 0.302–0.394 | <0.001 | −23 to +28 | 1,422 |

Note: Daily fever-clinic visits and influenza positivity series were smoothed using 7-day trailing moving averages. Lead–lag correlations were evaluated over the primary −28 to +28-day lag window. Negative lags indicate that influenza positivity preceded fever-clinic visits, whereas positive lags indicate that influenza positivity followed fever-clinic visits. Pearson correlation coefficients were calculated using lag-specific complete-case pairs. Confidence intervals were calculated using Fisher’s *z* transformation. *P* values were adjusted using the Benjamini–Hochberg false discovery rate method. CI, confidence interval; FDR, false discovery rate.

**Supplementary Table S3. Restricted regression analysis after exclusion of 2022**

| **Exposure** | **Lag** | **Seasonality-adjusted aIRR (95% CI)** | ***P value*** | **Testing-volume-adjusted aIRR (95% CI)** | ***P value*** | **N days** |
| --- | --- | --- | --- | --- | --- | --- |
| Overall influenza | 12-day lead | 1.181 (1.164–1.198) | <0.001 | 1.014 (0.993–1.035) | 0.196 | 1,092 |
| Influenza A | 15-day lead | 1.127 (1.109–1.145) | <0.001 | 0.984 (0.966–1.003) | 0.090 | 1,092 |
| Influenza B | lag 0 | 1.475 (1.424–1.528) | <0.001 | 1.124 (1.092–1.157) | <0.001 | 1,093 |

Note: Adjusted incidence rate ratios (aIRRs) were estimated using quasi-Poisson regression with daily fever-clinic visits as the outcome and are expressed per 10-percentage-point increase in influenza positivity. Seasonality-adjusted models included day of week, calendar month, and calendar year. Testing-volume-adjusted models additionally included lag-aligned log(1 + influenza testing episodes). The restricted analysis excluded all 2022 outcome observations and included outcome observations from 1 January 2023 through 31 December 2025. Exposure lags were retained from the primary analysis to assess robustness across study periods. Lagged predictors were generated from the continuous time series before restriction; therefore, late-2022 observations were used only where required to construct lagged exposures for early-2023 outcome dates and were not included as outcome observations. aIRR, adjusted incidence rate ratio; CI, confidence interval.

**Supplementary Table S4. Autocorrelation-robust inference for the fully adjusted 15-day influenza A model**

**Panel A. Autocorrelation-robust inference**

| **Analysis** | **aIRR*** | **95% CI** | ***P value*** | **N days** |
| --- | --- | --- | --- | --- |
| Original quasi-Poisson | 1.040 | 1.024–1.055 | <0.001 | 1,400 |
| HAC, 7-day bandwidth | 1.040 | 0.990–1.092 | 0.119 | 1,400 |
| HAC, 14-day bandwidth | 1.040 | 0.982–1.101 | 0.183 | 1,400 |
| HAC, 28-day bandwidth | 1.040 | 0.978–1.106 | 0.214 | 1,400 |

**Panel B. Residual autocorrelation diagnostics**

| **Diagnostic** | **Result** |
| --- | --- |
| Lag-1 Pearson residual correlation | 0.868 |
| Ljung–Box test, lag 7 | *P <0.001* |
| Ljung–Box test, lag 14 | *P <0.001* |
| Ljung–Box test, lag 28 | *P <0.001* |

*aIRR per 10-percentage-point increase in influenza A positivity at a 15-day lead. The primary fully adjusted influenza A model used the same mean specification as the testing-volume-adjusted model in Table 3, including day of week, calendar month, calendar year, and lag-aligned log(1 + influenza testing episodes). The original quasi-Poisson model used Pearson-scale adjustment for overdispersion. HAC/Newey–West covariance estimates were calculated using bandwidths of 7, 14, and 28 days. Pearson residual autocorrelation was assessed using the lag-1 correlation coefficient and Ljung–Box tests. aIRR, adjusted incidence rate ratio; CI, confidence interval; HAC, heteroskedasticity- and autocorrelation-consistent.

**Supplementary Table S5. Adjacent-lag sensitivity analysis of influenza A using HAC-robust inference**

**Panel A. Full study period, 2022–2025**

| **Lead, days** | **aIRR** | **HAC-adjusted 95% CI** | **HAC *P*** | **FDR-adjusted HAC *P*** | **N** |
| --- | --- | --- | --- | --- | --- |
| 0 | 0.938 | 0.888–0.991 | 0.021 | 0.294 | 1,415 |
| 1 | 0.945 | 0.897–0.996 | 0.035 | 0.294 | 1,414 |
| 2 | 0.958 | 0.911–1.008 | 0.101 | 0.294 | 1,413 |
| 3 | 0.966 | 0.920–1.014 | 0.161 | 0.294 | 1,412 |
| 4 | 0.973 | 0.929–1.018 | 0.240 | 0.330 | 1,411 |
| 5 | 0.984 | 0.942–1.027 | 0.456 | 0.528 | 1,410 |
| 6 | 0.992 | 0.952–1.033 | 0.698 | 0.731 | 1,409 |
| 7 | 0.998 | 0.959–1.039 | 0.912 | 0.912 | 1,408 |
| 8 | 1.009 | 0.970–1.050 | 0.662 | 0.728 | 1,407 |
| 9 | 1.016 | 0.977–1.057 | 0.430 | 0.525 | 1,406 |
| 10 | 1.023 | 0.982–1.066 | 0.279 | 0.360 | 1,405 |
| 11 | 1.028 | 0.984–1.074 | 0.217 | 0.318 | 1,404 |
| 12 | 1.031 | 0.985–1.079 | 0.187 | 0.294 | 1,403 |
| 13 | 1.035 | 0.987–1.085 | 0.161 | 0.294 | 1,402 |
| 14 | 1.035 | 0.985–1.086 | 0.172 | 0.294 | 1,401 |
| 15 | 1.040 | 0.990–1.092 | 0.119 | 0.294 | 1,400 |
| 16 | 1.047 | 0.997–1.099 | 0.067 | 0.294 | 1,399 |
| 17 | 1.046 | 0.995–1.099 | 0.077 | 0.294 | 1,398 |
| 18 | 1.043 | 0.992–1.096 | 0.098 | 0.294 | 1,397 |
| 19 | 1.039 | 0.989–1.091 | 0.125 | 0.294 | 1,396 |
| 20 | 1.037 | 0.990–1.086 | 0.129 | 0.294 | 1,395 |
| 21 | 1.031 | 0.986–1.078 | 0.185 | 0.294 | 1,394 |

**Panel B. Restricted period, 2023–2025**

| **Lead, days** | **aIRR** | **HAC-adjusted 95% CI** | **HAC *P*** | **FDR-adjusted HAC *P*** | **N** |
| --- | --- | --- | --- | --- | --- |
| 0 | 0.937 | 0.894–0.982 | 0.006 | 0.104 | 1,093 |
| 1 | 0.943 | 0.902–0.986 | 0.009 | 0.104 | 1,092 |
| 2 | 0.951 | 0.911–0.993 | 0.022 | 0.143 | 1,092 |
| 3 | 0.955 | 0.918–0.995 | 0.026 | 0.143 | 1,092 |
| 4 | 0.961 | 0.923–1.002 | 0.059 | 0.260 | 1,092 |
| 5 | 0.963 | 0.924–1.004 | 0.076 | 0.279 | 1,092 |
| 6 | 0.965 | 0.925–1.008 | 0.111 | 0.347 | 1,092 |
| 7 | 0.966 | 0.924–1.011 | 0.139 | 0.382 | 1,092 |
| 8 | 0.973 | 0.927–1.021 | 0.271 | 0.663 | 1,092 |
| 9 | 0.978 | 0.928–1.031 | 0.407 | 0.769 | 1,092 |
| 10 | 0.982 | 0.927–1.040 | 0.533 | 0.769 | 1,092 |
| 11 | 0.984 | 0.925–1.047 | 0.602 | 0.769 | 1,092 |
| 12 | 0.982 | 0.920–1.049 | 0.592 | 0.769 | 1,092 |
| 13 | 0.982 | 0.916–1.052 | 0.597 | 0.769 | 1,092 |
| 14 | 0.981 | 0.914–1.052 | 0.587 | 0.769 | 1,092 |
| 15 | 0.984 | 0.916–1.058 | 0.664 | 0.769 | 1,092 |
| 16 | 0.992 | 0.922–1.068 | 0.830 | 0.835 | 1,092 |
| 17 | 0.992 | 0.920–1.070 | 0.835 | 0.835 | 1,092 |
| 18 | 0.989 | 0.918–1.065 | 0.763 | 0.835 | 1,092 |
| 19 | 0.983 | 0.914–1.058 | 0.649 | 0.769 | 1,092 |
| 20 | 0.976 | 0.911–1.046 | 0.500 | 0.769 | 1,092 |
| 21 | 0.973 | 0.911–1.039 | 0.415 | 0.769 | 1,092 |

Note: aIRRs are expressed per 10-percentage-point increase in influenza A positivity at each indicated lead. All models included day of week, calendar month, calendar year, and lag-aligned log(1 + influenza testing episodes). HAC/Newey–West covariance estimates with a 7-day bandwidth were used for inference. FDR-adjusted *P* values were calculated within each panel across the 22 evaluated lead times from 0 to 21 days. Panel A includes 2022–2025; Panel B restricts outcome observations to 2023–2025. This analysis assessed temporal stability rather than identifying a uniquely optimal lag. aIRR, adjusted incidence rate ratio; CI, confidence interval; FDR, false discovery rate; HAC, heteroskedasticity- and autocorrelation-consistent.

Supplementary Table S6. Peak lead–lag correlations between influenza positivity and fever-clinic visits over the extended −56 to +56-day lag window

| **Influenza indicator** | **Peak lag, days** | **Peak correlation coefficient, r** | **95% CI at peak** | **FDR-adjusted *P* value** | **FDR-significant lag window** | **N pairs at peak** |
| --- | --- | --- | --- | --- | --- | --- |
| Overall influenza | −12 | 0.585 | 0.550–0.618 | <0.001 | −56 to +56 | 1,438 |
| Influenza A | −15 | 0.558 | 0.522–0.593 | <0.001 | −56 to +26 | 1,435 |
| Influenza B | +50 | 0.412 | 0.368–0.455 | <0.001 | −23 to +56 | 1,400 |

Note: Lag correlations were calculated using 7-day centered moving-average daily series over lags from −56 to +56 days. Negative lags indicate that influenza positivity preceded fever-clinic visits, whereas positive lags indicate that influenza positivity followed fever-clinic visits. Confidence intervals were calculated using Fisher’s *z* transformation. *P* values were adjusted separately within each influenza series across all 113 evaluated lags using the Benjamini–Hochberg FDR procedure. The FDR-significant lag window denotes the contiguous FDR-significant interval containing the maximum positive correlation. Because adjacent lagged observations are serially correlated, lag-specific inferential statistics should be interpreted descriptively. CI, confidence interval; FDR, false discovery rate.
